# Stages of development and injury: An epidemiological survey of young children presenting to an emergency department

**DOI:** 10.1186/1471-2458-8-120

**Published:** 2008-04-14

**Authors:** Kirsty MacInnes, David H Stone

**Affiliations:** 1 Faculty of Medicine, University of Glasgow, Glasgow, UK

## Abstract

**Background:**

The aim of our study was to use a local (Glasgow, west of Scotland) version of a Canadian injury surveillance programme (CHIRPP) to investigate the relationship between the developmental stage of young (pre-school) children, using age as a proxy, and the occurrence (incidence, nature, mechanism and location) of injuries presenting to a Scottish hospital emergency department, in an attempt to replicate the findings of a recent study in Kingston, Canada.

**Methods:**

We used the Glasgow CHIRPP data to perform two types of analyses. First, we calculated injury rates for that part of the hospital catchment area for which reasonably accurate population denominators were available. Second, we examined detailed injury patterns, in terms of the circumstances, mechanisms, location and types of injury. We compared our findings with those of the Kingston researchers.

**Results:**

A total of 17,793 injury records for children aged up to 7 years were identified over the period 1997–99. For 1997–2001, 6,188 were used to calculate rates in the west of the city only. Average annual age specific rates per 1000 children were highest in both males and females aged 12–35 months. Apart from the higher rates in Glasgow, the pattern of injuries, in terms of breakdown factors, mechanism, location, context, and nature of injury, were similar in Glasgow and Kingston.

**Conclusion:**

We replicated in Glasgow, UK, the findings of a Canadian study demonstrating a correlation between the pattern of childhood injuries and developmental stage. Future research should take account of the need to enhance statistical power and explore the interaction between age and potential confounding variables such as socio-economic deprivation. Our findings highlight the importance of designing injury prevention interventions that are appropriate for specific stages of development in children.

## Background

The close relationship between the stage of child development and the risk of injury is well recognised [1]. With each new stage of physical, cognitive and social development, new injury hazards emerge. Epidemiologically, this changing level of risk is reflected by the varying pattern of causes of injury across the age groups.

Although several formal health programmes aim to prevent injury in the early years, few population-based studies investigating the changing pattern of injury by developmental stage have been performed. By looking at patient demographics and details on the nature of injuries within small age groupings representative of developmental stage, it may be possible to identify population subgroups that could be at greater risk for injury. These insights are important for planning preventive strategies. One study carried out in Canada using the Kingston CHIRPP (Canadian Hospitals Injury Reporting and Prevention Programme) set out to address this gap in the literature [2]. They described changing mechanisms of injury according to developmental stage and used these findings to identify preventive priorities.

The aim of our study was to use a local version of CHIRPP [3] to investigate the relationship between the developmental stage of young (pre-school) children, using age as a proxy, and the occurrence (incidence, nature, mechanism and location) of injury, in an attempt to replicate the Kingston study and compare our findings with those of the Canadian researchers.

## Methods

### Glasgow CHIRPP

CHIRPP ran in the Royal Hospital for Sick Children (Yorkhill), Glasgow, from the mid-1990s to 2006. It was designed to capture information on all injuries presenting to the emergency department (ED) in order to generate data to assist injury prevention initiatives. A CHIRPP form was supposed to be completed for every injured child presenting at the ED. The first part was completed by the accompanying adult (or older child) and contained information on where the child was, what he/she was doing and what went wrong to cause the injury. The second part was completed by a clinician who provided details on the nature of the injury, the body parts involved and any treatment received. Once the CHIRPP form was completed the data were coded, entered and stored on a computer.

### Analyses of injury rates and patterns

We used the CHIRPP data to perform two types of analyses. First, we calculated injury rates for that part of the hospital catchment area for which reasonably accurate population denominators were available. Second, we examined detailed injury patterns, in terms of the circumstances, mechanisms, location and types of injury. Computerised data analysis was performed using SPSS v. 14 and Microsoft Excel 2007.

To calculate injury rates, we analysed the data for a period of five complete calendar years (1997 – 2001). In common with many other hospitals in Scotland, there was no rigidly enforced boundary for the Yorkhill catchment area. Cases from the west of Glasgow only were used in order to establish a clear denominator as it was assumed that most children injured in the west of the city would present to the Yorkhill ED, since they lived closest to the hospital. We adopted a denominator-based approach to the analysis as a previous study using the Glasgow CHIRPP showed that this method generated more reliable findings than a frequency based analysis [4].

Overall age and sex specific rates (per 1000 population) of injury and associated 95% confidence intervals were calculated for each of the five years of the study period. Ages were then re-coded allowing the data to be analysed in the four specified age categories. These were 0–11, 12–35, 36–59 and 60–83 months – the same categories used by the Kingston study [2] permitting comparison of the two sets of findings. The population at risk was considered to be all children under the age of seven years living in a west Glasgow postcode sector. **D**enominators were derived from 2001 census data that gave an estimated number of children living in each postcode sector in one-year age groupings.

To investigate injury patterns, we analysed the data for a period of three complete years (1997 – 1999). Later years were excluded from this part of the analysis as the CHIRPP collection forms were modified in 2000 to include tick boxes with the result that much of the detail surrounding the injury event was lost. All postcode sectors were included to increase the number of injury events available for analysis. The CHIRPP categories included "breakdown", "mechanism", "context" and "location". The categories describing the consequences of injury include "severest injury", "most severely injured body part" and "disposition". Multiple response tables and frequencies were formulated using SPSS to characterise injury patterns within and across age groups.

## Results

### Injury rates by age and sex categories

**Figure 1:**
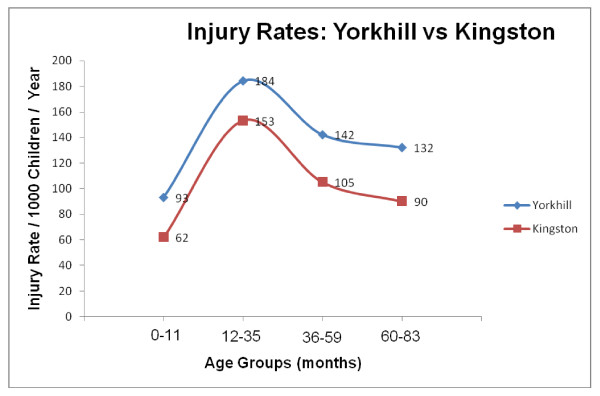
Trends in injury presentations to Yorkhill and Kingston by age. (Rates per 1000 population, calculated as an annual average over a 5 year period 1997–2001). CHIRPP data from west Glasgow postcode sectors only.

A total of 6,188 injury records of children under seven years, living in West Glasgow postcode sectors, were identified over the five-year study period. An average of 1,238 attended the Yorkhill ED each year, giving an average injury rate of 144/1000/year (95% CI 115 – 180). Average annual age specific rates per 1000 children were highest in both males and females aged 12–35 months (Table 1). Apart from the higher rates in Glasgow, the pattern followed a similar trend to that found in the Kingston study (Figure 1). Boys appeared at higher risk than girls in every age group though this was not statistically significant as the 95% confidence intervals consistently overlapped.

**Table 1:** Trends in injury presentations to Yorkhill ED by age category (rates per 1000 population, calculated as an annual average over a 5 year period 1997–2001). CHIRPP data from west Glasgow postcode sectors only.

	**Injury nos. and rates (per 1000 Child Years)**					
	
**Age Category (months)**	Total no. of Cases*	**Total rate**	**No. of cases: boys**	**Rates: boys**	**No. of cases: girls**	**Rates: girls**
**0–11**	592	**93 **(95%CI 63–138)	327	**105 **(95%CI 66 -166)	256	**79 **(95%CI 48 – 129)
**12–35**	2277	**184 **(95%CI 138–244)	1257	**192 **(95%CI 138–267)	975	**167 **(95%CI 118 – 237)
**36–59**	1717	**142 **(95%CI 105–192)	1018	**163 **(95%CI 115–231)	668	**114 **(95%CI 78 – 168)
**60–83**	1602	**132 **(95%CI 97–180)	922	**146 **(95%CI 102–208)	648	**111 **(95%CI 75 – 164)
**Total**	6188	**144 **(95%CI 115–180)	3524	**159 **(95%CI 123–205)	2547	**123 **(95%CI 93 – 162)

*These include some cases for which sex of child was unrecorded

### Pattern of Injury Analysis

We identified, through CHIRPP, a total of 17,793 children aged up to seven years, living in all Glasgow postcode sectors, who attended the emergency department of the hospital with an injury or ingestion over the study period (1997–1999).

#### "Breakdown" and "mechanism" factors

The details of what went wrong ("breakdown") to cause the injury and what actually inflicted the injury ("mechanism") can be seen in Tables 2 and 3 respectively. Falls were responsible for 41% of all injuries. Drops and falls were highest in the 0–11 month category where they accounted for 55% of all injuries in this age group. They declined to 39% in the 12–35 month age group and then levelled off. Other relatively frequent types of injury were ingestions (61% of which were in toddlers aged 12–35 months), strains, grazes/lacerations (that became progressively more frequent with increasing age), and foreign body injuries (that peaked in frequency at 36–59 months).

**Table 2:** Trends in breakdown factors (what went wrong) resulting in the injury at presentation to Yorkhill ED by age category. (Frequencies are an annual average of injury types in each age group over a 3 year period 1997–1999).

**Breakdown/Age Category**	**1 (0–11 months)**		**2 (12–35 months)**		**3 (36–59 months)**		**4 (60–83 months)**	
	
	No.	**%**	No.	**%**	No.	**%**	No.	**%**
Dropped/fell	1080	**54.5%**	2805	**39.1%**	1781	**38.5%**	1579	**39.3%**
Person/thing in dangerous position	373	**18.8%**	1759	**24.5%**	1006	**21.8%**	661	**16.5%**
Strained/over exerted	236	**11.9%**	1031	**14.4%**	760	**16.5%**	714	**17.8%**
Tripped/slipped	89	**4.5%**	900	**12.5%**	583	**12.6%**	534	**13.3%**
Caught in/snagged by object	20	**1.0%**	208	**2.9%**	150	**3.2%**	100	**2.5%**
Knocked over/spilt something	61	**3.1%**	153	**2.1%**	36	**0.8%**	25	**0.6%**
Horseplay	31	**1.6%**	77	**1.1%**	103	**2.2%**	132	**3.3%**
Fight	18	**0.9%**	59	**0.8%**	67	**1.5%**	91	**2.3%**
Collision with/hit by or against object	5	**0.3%**	3	**0.0%**	6	**0.1%**	12	**0.3%**
Other	69	**3.5%**	176	**2.5%**	126	**2.7%**	167	**4.2%**
Missing	1	**0.1%**	3	**0.0%**	2	**0.0%**	1	**0.0%**
**Total**	**1983**	**100%**	**7174**	**100%**	**4620**	**100%**	**4016**	**100%**

**Table 3:** Trends in mechanism (how the injury was inflicted) by which the injury was inflicted at presentation to Yorkhill ED by age. (Frequencies are an annual average of injury types in each age group over a 3 year period 1997–1999).

**Mechanism/Age Category**	**1 (0–11 months)**		**2 (12–35 months)**		**3 (36–59 months)**		**4 (60–83 months)**	
	
	No.	**%**	No.	**%**	No.	**%**	No.	**%**
Collision with/hit by or against object	1466	**73.9%**	4418	**61.6%**	3149	**68.2%**	3012	**75.0%**
Ingested/inhaled hazard	114	**5.7%**	824	**11.5%**	305	**6.6%**	109	**2.7%**
Strained/over exerted	104	**5.2%**	537	**7.5%**	288	**6.2%**	188	**4.7%**
Grazed/lacerated	58	**2.9%**	302	**4.2%**	247	**5.3%**	274	**6.8%**
Foreign body	36	**1.8%**	252	**3.5%**	242	**5.2%**	143	**3.6%**
Caught in/snagged by object	34	**1.7%**	325	**4.5%**	221	**4.8%**	157	**3.9%**
Other	163	**8.2%**	494	**6.9%**	162	**3.5%**	124	**3.1%**
Missing	8	**0.4%**	22	**0.3%**	6	**0.1%**	9	**0.2%**
**Total**	**1983**	**100%**	**7174**	**100%**	**4620**	**100%**	**4016**	**100%**

#### Location of injury occurrence

Over two-thirds (68%) of injuries occurred in home locations. The number of injuries occurring in the home decreased with advancing age, from 85% in the 0–11 month age group to only 45% in the 60–83 month group. Injuries in educational facilities, footpaths, playgrounds and sports facilities became more frequent with increasing age (Table 4).

**Table 4:** Trends in location (where injury happened) where the injury occurred at presentation to Yorkhill ED by age. (Frequencies are an annual average of injury type in each age group over a 3 year period 1997–1999).

**Location/Age Category**	**1 (0–11 months)**		**2 (12–35 months)**		**3 (36–59 months)**		**4 (60–83 months)**	
	
	No.	**%**	No.	**%**	No.	**%**	No.	**%**
Home	1686	**85.0%**	5745	**80.1%**	2897	**62.7%**	1811	**45.1%**
Footpath	74	**3.7%**	349	**4.9%**	546	**11.8%**	713	**17.8%**
Educational facility	12	**0.6%**	13	**0.2%**	25	**0.5%**	153	**3.8%**
Playground	26	**1.3%**	191	**2.7%**	245	**5.3%**	651	**16.2%**
Road/parking area	36	**1.8%**	112	**1.6%**	178	**3.9%**	200	**5.0%**
Sports facility	7	**0.4%**	27	**0.4%**	52	**1.1%**	68	**1.7%**
Other	99	**5.0%**	596	**8.3%**	545	**11.8%**	281	**7.0%**
Missing	43	**2.2%**	141	**2.0%**	132	**2.9%**	139	**3.5%**
**Total**	**1983**	**100%**	**7174**	**100%**	**4620**	**100%**	**4016**	**100%**

#### Context of injury

Almost two-thirds (62%) of injuries happened when the child was engaged in play. This proportion was lowest in the 0–11 month age category (37%), was almost double (64) % in the 12–35 month category and thereafter levelled off.

Around 17% of injuries took place when the child was on the move (walking, running or crawling). This proportion was lowest in the 0–11 month age category (10%) and almost doubled (19%) in the 12–35 month category where it reached a plateau and thereafter remained steady (Table 5).

**Table 5:** Trends in context (what child was doing at time of injury) surrounding the injury at presentation to Yorkhill ED by age. (Frequencies are an annual average of injury type in each age group over a 3 year period 1997–1999).

**Context/Age Category**	**1 (0–11 months)**		**2 (12–35 months)**		**3 (36–59 months)**		**4 (60–83 months)**	
	
	No.	**%**	No.	**%**	No.	**%**	No.	**%**
Walking/running/crawling	199	**10.0%**	1342	**18.7%**	832	**18.0%**	705	**17.6%**
Playing	731	**36.9%**	4602	**64.1%**	3027	**65.5%**	2654	**66.1%**
Riding bicycle/scooter	6	**0.3%**	34	**0.5%**	123	**2.7%**	197	**4.9%**
Sitting	430	**21.7%**	379	**5.3%**	156	**3.4%**	101	**2.5%**
Household activity/personal hygiene	76	**3.8%**	192	**2.7%**	111	**2.4%**	80	**2.0%**
Standing	48	**2.4%**	254	**3.5%**	146	**3.2%**	90	**2.2%**
Passenger in vehicle	25	**1.3%**	44	**0.6%**	49	**1.1%**	38	**0.9%**
Playing sports	3	**0.2%**	10	**0.1%**	23	**0.5%**	47	**1.2%**
Other	329	**16.6%**	176	**2.5%**	70	**1.5%**	40	**1.0%**
Missing/invalid	136	**6.9%**	139	**1.9%**	83	**1.8%**	63	**1.6%**
**Total**	**1983**	**100%**	**7174**	**100%**	**4620**	**100%**	**4016**	**100%**

#### Anatomical site of injury

There were substantial variations in the most frequent types of injury by age group. The head was involved in 52% of injuries in the 0–11 month age group but this declined with age and comprised only 44% of all injuries in the 60–83 month age group. Injuries affecting the extremities showed the opposite trend with those affecting the upper extremity steadily increasing from 10% in the 0–11 month age group to 20% in the 60–83 month age group. Injuries affecting the lower extremities also rose, from 8% in the 0–11 month age group to 21% in the 60–83 month group.

#### Nature of injury

Open wounds and fractures increased progressively with age, with open wounds rising from 10% of all injuries in the 0–11 month age group to 27% in the 60–83 month group. Fractures increased from 6% of all injuries in the youngest group to 17% in the eldest group.

## Discussion

Remarkably little epidemiological research has focused on the changing risk of injury in young children according to developmental stage. To our knowledge, our study is the first to investigate this issue using a population-based approach in a high-risk area of the UK. Moreover, we were able to compare our experience in the West of Scotland with that of Canadian colleagues, thanks to the operation in both locations of a virtually identical injury surveillance system.

### Injury Rates by Age Category

The pattern of injury rates at Yorkhill displayed a similar pattern across the age groups to the Kingston data. This consistency offers generalisable evidence of a relationship between stage of development and injury risk. The finding is a plausible one. Once mobility is established, exploration of their surroundings is a crucial part of childhood development but it is a behaviour that, combined with their lack of understanding of – and physiological incapacity to negotiate – many environmental hazards, places them at particular risk of injury [2].

The average annual injury rate in Yorkhill was higher than that of Kingston though the difference was not quite statistically significant. A higher rate at Yorkhill may reflect different degrees of socio-economic deprivation in the two populations and injury risk is correlated with poverty. Even within the UK, Scotland exhibits significantly higher rates of injury than in England and Wales [5], probably for that reason.

As in Kingston, the average annual rate at Yorkhill was higher for boys than for girls, though this did not reach statistical significance. A number of factors may contribute to the male excess in injury mortality, including differences in exposure to hazards, behaviour and socialisation as well as possible differences in parental attitudes to boys and girls.

### Patterns of Injury

The observed injury patterns reflect the exposure of young children to a range of hazards, with varying and continuously changing vulnerabilities over time that are influenced by physical, cognitive and social characteristics at different stages of development.

#### Mechanisms of injury

The finding that drops and falls were the most common causes of injury and peaked in the first year was consistent with the Kingston study. This is unsurprising as during this time children become mobile and increasingly curious about their environment. Ingestions and foreign body injuries were also fairly common cause of injury and most numerous in the 12–35 month age category (61%). This was similar to the Canadian data and may be explained by the developmental progress that occurs during this period of a child's life.

#### Location of injury occurrence

The home was the most common place for an injury to occur in all age groups, particularly in the first year. This result too is unsurprising and reflects the amount of time a young child spends in the home, usually in the presence of a carer. Injuries outside of the home in places such as playgrounds, footpaths, sports and educational facilities all increased with age. This may be attributable to the gradual emergence of the child from the home and a growing attempt to achieve independence from adult carers.

#### Nature of injury

The most common type of injury was a blow to the head and this was highest in the 0–11 month group (52%). This was also reported in the Kingston study and may be due to two factors: the minimal control that babies are able to exert over head position and movements, and their relatively inability to take avoiding or protective action during a fall or when confronted with an external hazard. Injuries affecting the upper limb displayed the second highest frequency and peaked in the 60–83 month group. This is probably because older children tend to throw up their hands to protect their head when they fall, thus placing their arms at increased risk of injury.

### Strengths and weaknesses of the study

The main disadvantage of using Yorkhill as a setting for an ED based surveillance system was the lack of a clear denominator. As with other hospitals in Glasgow, there is no clearly delineated catchment area. By including only cases from the West of the city, we are reasonably confident of their representative nature for the purpose calculating rates. At the same time, we inevitably sacrificed a larger potential sample size and therefore statistical power.

As the injury rates calculated were limited to ED encounters, they are likely to underestimate the actual burden of paediatric injury in the population. In effect, we examined only the tip of the injury iceberg because a proportion (perhaps as many as two-thirds) of all injuries present to primary care [6]. Furthermore, CHIRPP relied on self-reports, the reliability of which is a matter for speculation.

One area of concern about the Yorkhill data was the frequent lack distinction drawn between the data coded in the 'mechanism' and 'breakdown' variables. Another related to the field for intent as this was often left incomplete, possibly due to staff reservations about possible medico-legal consequences.

Ideally, an injury surveillance system should capture information on the severity of injury, or at least include only those cases that exceed a predetermined severity threshold [7]. At Yorkhill, as at other CHIRPP sites, such information was not included.

Data collection rates varied depending on the time of day and the day of the week. By confining the study period to the selected years, we attempted to minimise the impact of such varying data quality; in the period 1997 to 2001, the capture and completion rates were known to be consistently high (over 90%).

Despite its limitations, our study employed a population-based approach using an evaluated injury surveillance system [8] to investigate a large number of injuries to children residing in a busy emergency department located in a relatively high risk urban area. We believe that our findings are therefore scientifically robust and have generated important insights into the link between the development of young children and their risk of injury. These insights should prove as valuable to those responsible for planning and implementing preventive measures in Glasgow as they have in Kingston. The key developmentally-sensitive preventive priorities identified by the Kingston group – careful supervision and risk anticipation, limiting access to hazards, and fall avoidance through safe play behaviour and environments – are all equally applicable in Glasgow and, most probably, elsewhere.

## Conclusion

We replicated the key findings of the Kingston study that there is a clear correlation between the developmental stage of children and the injuries they sustain, thereby reinforcing the pertinence of the preventive messages offered by our Canadian colleagues. Future research should take account of the need to enhance statistical power and explore the interaction between age and potential confounding variables such as socio-economic deprivation. Our findings highlight the importance of designing injury prevention interventions that are appropriate for specific stages of development in children.

## Competing interests

The author(s) declare that they have no competing interests.

DS conceived the study, advised on its design, supervised the analysis and assisted with the writing of the paper. KM conducted the study, undertook the analysis and was the main contributor to the writing of the paper.

## Pre-publication history

The pre-publication history for this paper can be accessed here:


